# An integrated global chemomics and system biology approach to analyze the mechanisms of the traditional Chinese medicinal preparation *Eriobotrya japonica – Fritillaria usuriensis* dropping pills for pulmonary diseases

**DOI:** 10.1186/s12906-015-0983-y

**Published:** 2016-01-08

**Authors:** Jin Tao, Yuanyuan Hou, Xiaoyao Ma, Dan Liu, Yongling Tong, Hong Zhou, Jie Gao, Gang Bai

**Affiliations:** 1 State Key Laboratory of Medicinal Chemical Biology and College of Pharmacy, Nankai University, Tianjin, 300071 People’s Republic of China; 2 Tianjin Key Laboratory of Molecular Drug Research, Nankai University, Tianjin, 300071 People’s Republic of China; 3 No.6 TCM Factory, Zhongxin Pharmaceuticals, Tianjin, 300193 People’s Republic of China

**Keywords:** UPLC/Q-TOF-MS, Molecular docking, RNA-Sequence, Network pharmacology, Airway inflammation, *Eriobotrya japonica – Fritillaria usuriensis* dropping pills

## Abstract

**Background:**

Traditional Chinese medicine (TCM) herbal formulae provide valuable therapeutic strategies. However, the active ingredients and mechanisms of action remain unclear for most of these formulae. Therefore, the identification of complex mechanisms is a major challenge in TCM research.

**Methods:**

This study used a network pharmacology approach to clarify the anti-inflammatory and cough suppressing mechanisms of the Chinese medicinal preparation *Eriobotrya japonica – Fritillaria usuriensis* dropping pills (ChuanbeiPipa dropping pills, CBPP). The chemical constituents of CBPP were identified by high-quality ultra-performance liquid chromatography/quadrupole time-of-flight mass spectrometry (UPLC/Q-TOF-MS), and anti-inflammatory ingredients were selected and analyzed using the PharmMapper and Kyoto Encyclopedia of Genes and Genomes (KEGG) bioinformatics websites to predict the target proteins and related pathways, respectively. Then, an RNA-sequencing (RNA-Seq) analysis was carried out to investigate the different expression of genes in the lung tissue of rats with chronic bronchitis.

**Results:**

Six main constituents affected 19 predicted pathways, including ursolic acid and oleanolic acid from *Eriobotrya japonica* (Thunb.) Lindl. (***Eri***), peiminine from *Fritillaria usuriensis* Maxim. (***Fri***), platycodigenin and polygalacic acid from *Platycodon grandiflorum* (Jacq.) A. DC. (***Pla***) and guanosine from *Pinellia ternata* (Thunb.) Makino. (***Pin***). Expression of 34 genes was significantly decreased after CBPP treatment, affecting four therapeutic functions: immunoregulation, anti-inflammation, collagen formation and muscle contraction.

**Conclusion:**

The active components acted on the mitogen activated protein kinase (MAPK) pathway, transforming growth factor (TGF)-beta pathway, focal adhesion, tight junctions and the action cytoskeleton to exert anti-inflammatory effects, resolve phlegm, and relieve cough. This novel approach of global chemomics-integrated systems biology represents an effective and accurate strategy for the study of TCM with multiple components and multiple target mechanisms.

**Electronic supplementary material:**

The online version of this article (doi:10.1186/s12906-015-0983-y) contains supplementary material, which is available to authorized users.

## Background

A traditional Chinese medicine (TCM) preparation contains various active ingredients that exhibit synergistic effects on multiple targets to treat disease. However, the potential mechanisms of action are difficult to research systematically. Recently, network modeling and network analysis technology have been proven useful for obtaining gene and protein information and for identifying potential targets and pathways [1]. The development of analytical theory, such as systems biology and network pharmacology, provides an opportunity to clarify the complex and holistic mechanisms by which TCM treats complex diseases [2].

Liang et al. obtained a better understanding of the network pharmacology analysis of the herbal formula Liuwei Dihuang [3]. The chemical constituents of Liuwei Dihuang with high drug-likeness were selected, and the related targets and pathway were predicted. Based on the predicted drug-target-disease network, 6 compounds and 4 targets were verified. In another study, the pharmacological mechanism of Si-Wu-Tang was explored using multi-level data integration [4]. The gene expression of MCF-7 cells treated with Si-Wu-Tang was evaluated by microarray, and the potential targets of each herb were identified using the TCM Integrative Database. Using the results, a herb-ingredient-target-drug was constructed for gynecological disease treatment. Zhao et al. regulated the Qingfei Xiaoyan Wan to alleviate asthma through a multi-target network [1]. Differentially expressed genes and proteins in lung tissue were detected and analyzed using the gene ontology (GO) terms in the Kyoto Encyclopedia of Genes and Genomes (KEGG). Li et al. studied Qishen Yiqi to reveal its underlying multi-compound, multi-target, multi-pathway mode of action [5]. The differentially expressed genes were identified from a myocardial infarction rat model treated with Qishen Yiqi, and their functions were analyzed based on the cardiovascular disease-related literature. Most of the above operations of TCM network pharmacology were performed virtually, and neither trace ingredients nor drug absorption were reflected at the biological level. The virtual evaluation presented a huge and complex network in which the main contact between compounds, targets and function was not clear, and it was not possible to conduct further studies on key issues.

TCM has been used to treat chronic airway disease for thousand years. Chronic bronchitis consists of airway inflammation, mucus secretion, and airway muscle contraction. The inflammatory response was shown to be dependent on a network of cytokines and chemokines interactions, including tumor necrosis factor (TNF)-α, interleukin (IL)-6, IL-8 and IL-1β [6]. Considering the complexity of this network, the main strategy was multi-targeting treatment. *Eriobotrya japonica – Fritillaria usuriensis* dropping pills (ChuanbeiPipa dropping pills, CBPP), which have been used in the clinical treatment of pulmonary diseases, is a modified form of a famous ancient formula consisting of *Eriobotrya japonica* (Thunb.) Lindl. (***Eri***), *Fritillaria usuriensis* Maxim. (***Fri***), *Platycodon grandiflorum* (Jacq.) A. DC. (***Pla***), *Pinellia ternata* (Thunb.) Makino. (***Pin***) and volatile oil extracts from *Mentha haplocalyx* Briq. (***Men*** **)**. In clinical practice, CBPP had good clinical therapeutic effects for eliminating cough, phlegm, chronic bronchitis and inflammation of the lungs.

In this study, we developed an integrated network pharmacological approach combining the virtual prediction of targets and pathways with the experimentally determined differences in gene expression to explain the synergistic mechanism of CBPP. After high-quality ultra-performance liquid chromatography (UPLC) separation, ingredients of CBPP were analyzed using quadrupole time-of-flight mass spectrometry (Q-TOF-MS) to obtain structural information. Representative compounds were selected and their targets and pathways were analyzed using PharmMapper and KEGG, respectively. Concomitantly, the expression of functional genes was determined using RNA-Sequencing (RNA-Seq)-based transcriptome analysis of the lung tissue from rats with chronic bronchitis. An integrated analysis was used to clarify the relationship between the major active components, the affected targets and affected pathways.

## Methods

### Plant materials

CBPP (Lot No. 633003) and its extract were donated by No.6 TCM Factory of Zhongxin Pharmaceuticals (Tianjin, China). The quality of each herb and CBPP extract were verified by marker compounds. Quantitative methods and results are provided in the Additional file 1.

### Chemicals and reagents

UPLC-grade acetonitrile and acetic acid were purchased from Merck (Darmstadt, Germany). Deionized water was purified using the Milli-Q system (Millipore, Bedford, MA, USA). Lipopolysaccharides (LPS) were obtained from Sigma Corporation (St. Louis, USA). Bacillus calmette-guerin (BCG) injections were purchased from Siqi Biological Pharmaceutical Corporation (Hunan China, Lot No. 130631). Dexamethasone (Dex) tablets were purchased from Xianju Pharmaceutical Corporation (Zhejiang China, Lot No. 131223).

### UPLC/Q-TOF-MS analysis

A UPLC System (Waters, USA) equipped with a photodiode array detector was used. An Acquity BEH C18 column (2.1 × 100 mm, 1.7 μm; Waters, USA) was used for the separation. The injection volume was 5 μL at a concentration of 10 mg/mL CBPP extract. The mobile phase consisted of 0.1 % (v/v) formic acid solution (A) and acetonitrile (B) at a flow rate of 0.4 mL/min. A gradient program was used as follows: 0 min, 2 % B; 13 min, 30 % B; 16 min, 50 % B; 25 min, 80 % B; 28 min, 100 % B; 28–30 min, 100 % B. The column temperature was 30 °C.

Accurate mass measurements were collected using a Q-TOF Premier with an electrospray ionization system (Waters, USA). The electrospray capillary voltage was 3.0 kV and 2.5 kV for the positive and negative modes, respectively. The sample cone voltage was 30 V. The nebulization gas was 600 L/h at 350 °C. The cone gas was 50 L/h, and the source temperature was 110 °C. The Q-TOF Premier acquisition rate was 0.1 s, with a 0.02 s inter-scan delay. The instrument was operated with the first resolving quadrupole in a wide pass mode (50 – 2,500 Da).

### Anti-inflammatory target prediction and docking

Candidate ingredients with anti-inflammatory activity or the main components that have been reported for the four herbs were selected, and their structures were put into the PharmMapper database (http://59.78.96.61/pharmmapper/) for target prediction. Pathway analysis was carried out using the KEGG website (http://www.genome.jp/kegg/).

To further evaluate the selectivity, candidate molecules were optimized using SYBYL X2.0 and then docked to targets. The crystal structures of potential target proteins were obtained from the Protein Data Bank (ID: 3EQF, 3V6R, 3KJF, 3LDX, 4KXZ, 4EFL and 3CU8). PDBQT-format files of the targets and ligands were prepared using AutoDockTools. 30 genetic algorithm (GA) runs were used to simulate ligand-receptor binding. The step size parameters of quaternion and torsion were set to 30. For each compound, 30 independent runs were performed. The default values were used for the other parameters.

### Ethics statement

Animal treatment and maintenance were performed in accordance with the Principle of Laboratory Animal Care (NIH Publication no. 85–23, revised 1985), and the Animal Ethics Committee of Nankai University approved the experimental protocol.

### LPS-induced chronic bronchitis in rats

Sprague-Dawley (SD) rats weighing 140–160 g were purchased from the Experimental Animal Center of the National Institute for the Control of Pharmaceutical and Biological Products (Beijing, China), and the batch number was 0006407. All of the animals were housed at a temperature of 21–23 °C, a relative humidity of 40–60 %, and a photoperiod of 12 h light-12 h dark. The animals had free access to food and water. They were allowed 3 days to adjust to the facilities before experimentation. Chronic bronchitis model was induced by BCG treatment (2.5 mL/kg) through the caudal vein, and LPS treatment (1 mg/kg) through endotracheal instillation 1 week later [7]. Then, the animals were randomly divided into six groups (*n* = 10): the control group (Con), the model group (Mod), the Dex group, and the high, middle, and low CBPP dose groups (CBPP-H, CBPP-M, CBPP-L). One week after LPS injection, drugs were given to each group continuously for 2 weeks. The treatment groups received CBPP at one of three doses (30, 60, or 120 mg/kg daily) or Dex (1.2 mg/kg daily). The Con and Mod received normal saline.

Blood samples were drawn from the abdominal aorta of anaesthetized rats. The serum supernatant was stored at -20 °C for subsequent testing. Then, the animals were euthanized. Bronchoalveolar lavage fluid (BALF) was prepared by washing the left lung three times with 10 mL of phosphate-buffered saline (PBS). The BALF was centrifuged at 1,500 rpm for 10 min, and the supernatant was collected and stored at -20 °C for cytokine analysis. The cell pellets were resuspended in PBS (1 mL), and the leukocyte counts were determined using a hemocytometer. Concentrations of TNF-α and IL-8 in the serum and BALF were measured using ELISA kits (Pierce/Endogen, Rockford). The left lung was rapidly stored at -80 °C for future RNA-Seq analysis. The right lung was fixed in 4 % paraformaldehyde for hematoxylin and eosin (H&E) staining.

### RNA-Seq analysis

Three lung samples, including Con, Mod and CBPP-M, were selected for RNA-Seq-based transcriptome analysis that was entrusted to Beijing Genomics Institute-Shenzhen. The total RNA of lungs was sequenced via Illumina HiSeqTM 2000 to obtain raw reads. Genes were screened by comparison with the reference genome. The functional annotation of differentially expressed genes was obtained from a GO and pathway enrichment analysis.

The screened genes of the three samples were compared to each other, including Con *vs* Mod, Con *vs* CBPP-M and Mod *vs* CBPP-M. The Log_2_Ratio is the division value of the differential expression quantity of two groups. Cluster analysis was implemented with MATLAB2011a (MathWorks, USA) using Euclidean distance and words connection. The protein interaction with corresponding genes was analyzed using the String 9.1 website (http://string-db.org/).

### Statistical analysis

The results were presented as the mean ± standard error (SEM). A statistical evaluation of multiple comparisons of the data was performed using one-way analysis of variance (ANOVA) followed by Bonferroni’s post hoc test. For single comparison, the significance between groups was determined using Student’s *t* test. The statistical significance was set *p* < 0.05.

## Results and discussion

### Composition analysis and active ingredient identification during CBPP extraction

**Fig. 1:**
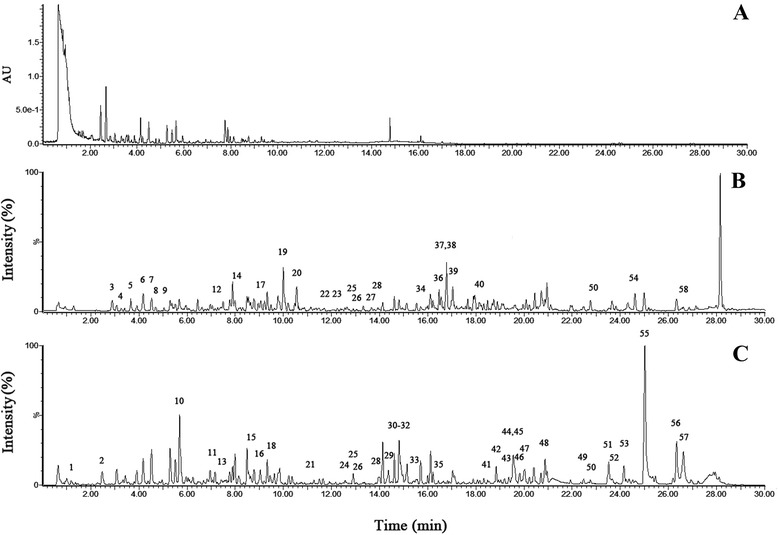
UPLC/Q-TOF-MS analysis of CBPP extracts. **a** UPLC/UV chromatograms of the extracts; **b**, **c** TIC chromatograms in positive ESI mode and negative ESI mode, respectively

The optimal UPLC/Q-TOF-MS method was used to characterize CBPP extracts. The UPLC/diode array detection analysis is shown in Fig. 1a. The total ion current chromatograms in the positive and negative modes are shown in Fig. 1b and c, respectively. The compositions of all of the constituents were deduced based on their molecular weight and reference, and 58 compounds were identified in total (Additional file 2: Table S1).

**Fig. 2:**
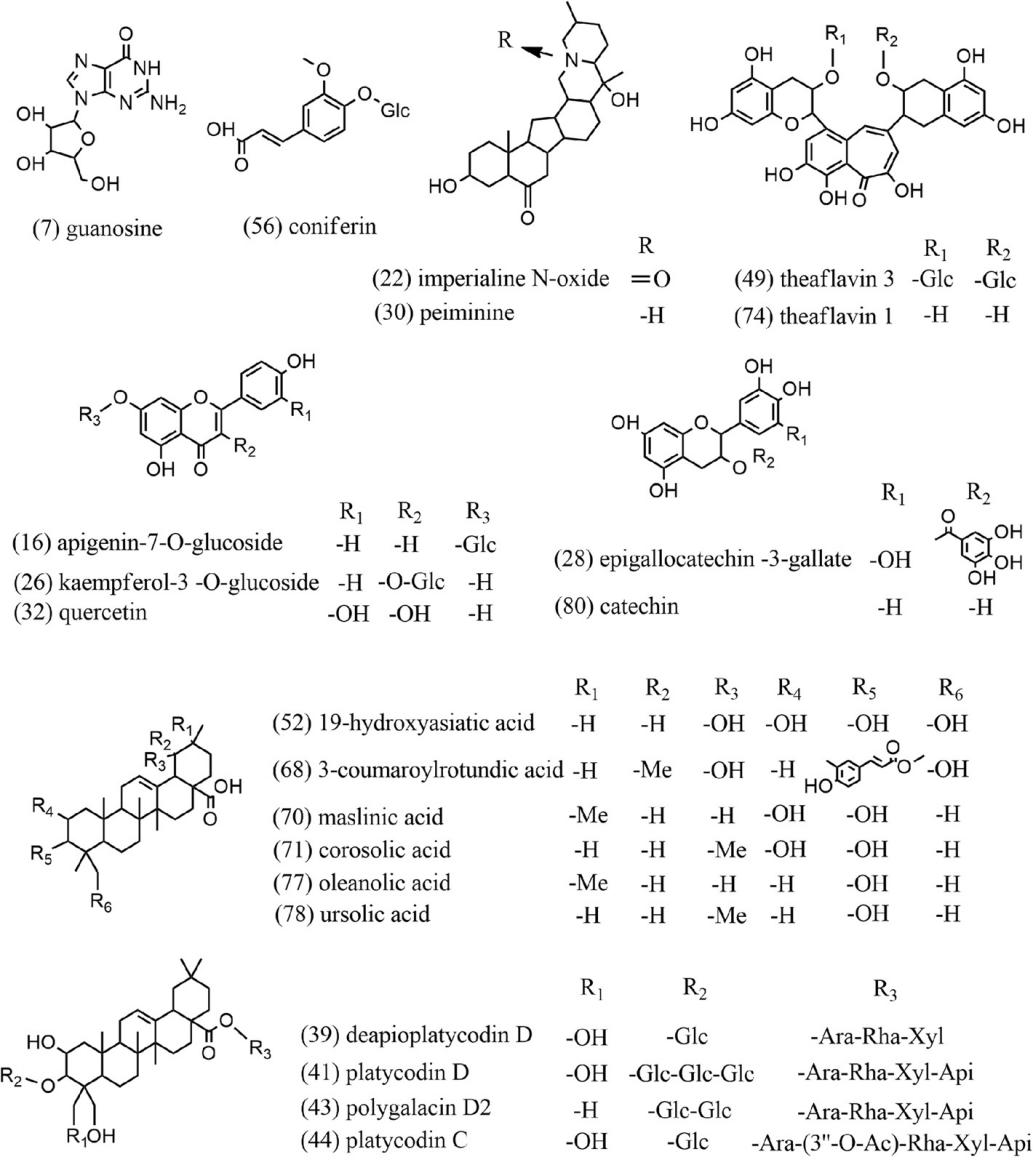
Chemical structures of the CBPP extract components with anti-inflammatory activity

Among the 58 compounds, 21 ingredients had anti-inflammatory activity in previous reports, and detailed information is presented in Fig. 2. The triterpene acid derivatives were the main active ingredient in ***Eri***, including four of the ursane-type and two of the oleanane-type compounds [8, 9]. There were five flavone derivatives and two theaflavin derivatives in ***Eri*** [10, 11], and two peiminine derivatives were identified in ***Fri*** [12, 13]. Oleanan esaponin derivatives were derived from ***Pla***, including three platycodin and one polygalacin compounds [14, 15]. The active ingredients in ***Pin*** included guanosine and coniferin [16, 17].

### LPS-induced chronic bronchitis reduced by CBPP in rats

**Fig. 3:**
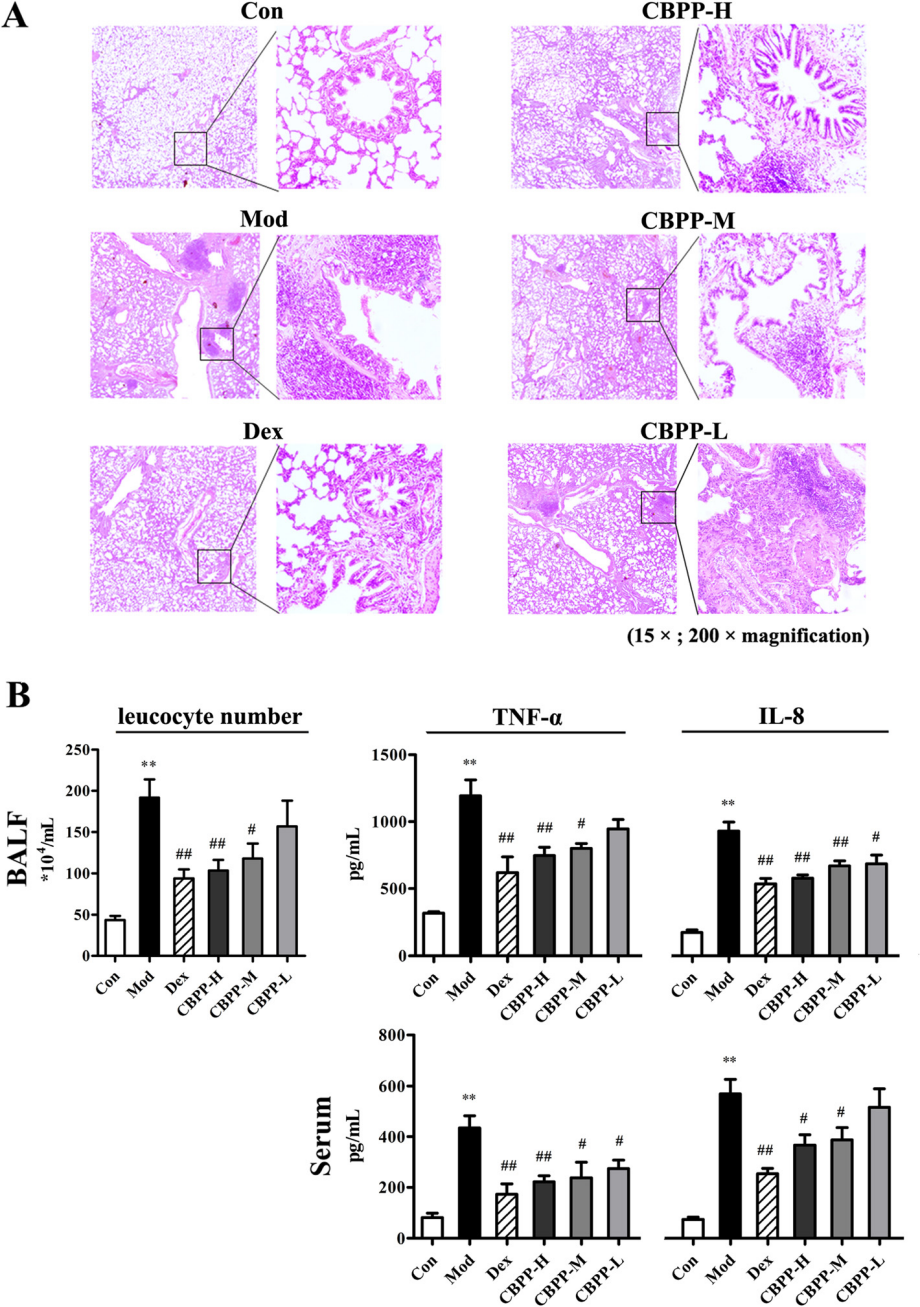
CBPP alleviated LPS-induced chronic bronchitis in rats. **a** H&E staining images of rat lungs; **b** Leukocyte numbers in BALF and the ELISA of the inflammatory cytokines TNF-α and IL-8 in BALF and serum. ^*^ *p* < 0.05 and ^**^ *p* < 0.01 compared to the Con. ^#^ *p* < 0.05 and ^##^ *p* < 0.01, compared to the Mod (*n* = 10)

To evaluate the effects of CBPP treatment on LPS-induced chronic bronchitis in rats, histological changes and inflammatory cytokines were observed. As shown in Fig. 3a, compared to the structural integrity of the lung tissue in the Con group, LPS infection caused capillary congestion, the obstruction of small airways by lymphocytic infiltrates, and a widening of the alveolar septa. Treatment with CBPP significantly reduced the histologically detectable injury, reducing the obstruction of small airways and the recruitment of inflammatory infiltrates. The number of leukocytes in the BALF is an important index for the evaluation of lung inflammation. Compared to the Con group, the number of leukocytes was four times greater in the Mod group. However, the decrease in leukocytes in the groups treated with the CBPP varied in a dose-dependent manner. These data indicated that CBPP prevented the excessive infiltration of leukocytes into the lung tissues. After LPS challenge, the concentrations of typical cytokines, such as TNF-α and IL-8, were measured in the serum and BALF (Fig. 3b). CBPP-L did not prevent the release of IL-8 in the serum or of TNF-α in BALF; however, treatment with higher doses of CBPP decreased the production of these cytokines to varying degrees. These results agreed with the histological changes that were assessed above.

### Target and pathway prediction of active ingredients

**Fig. 4:**
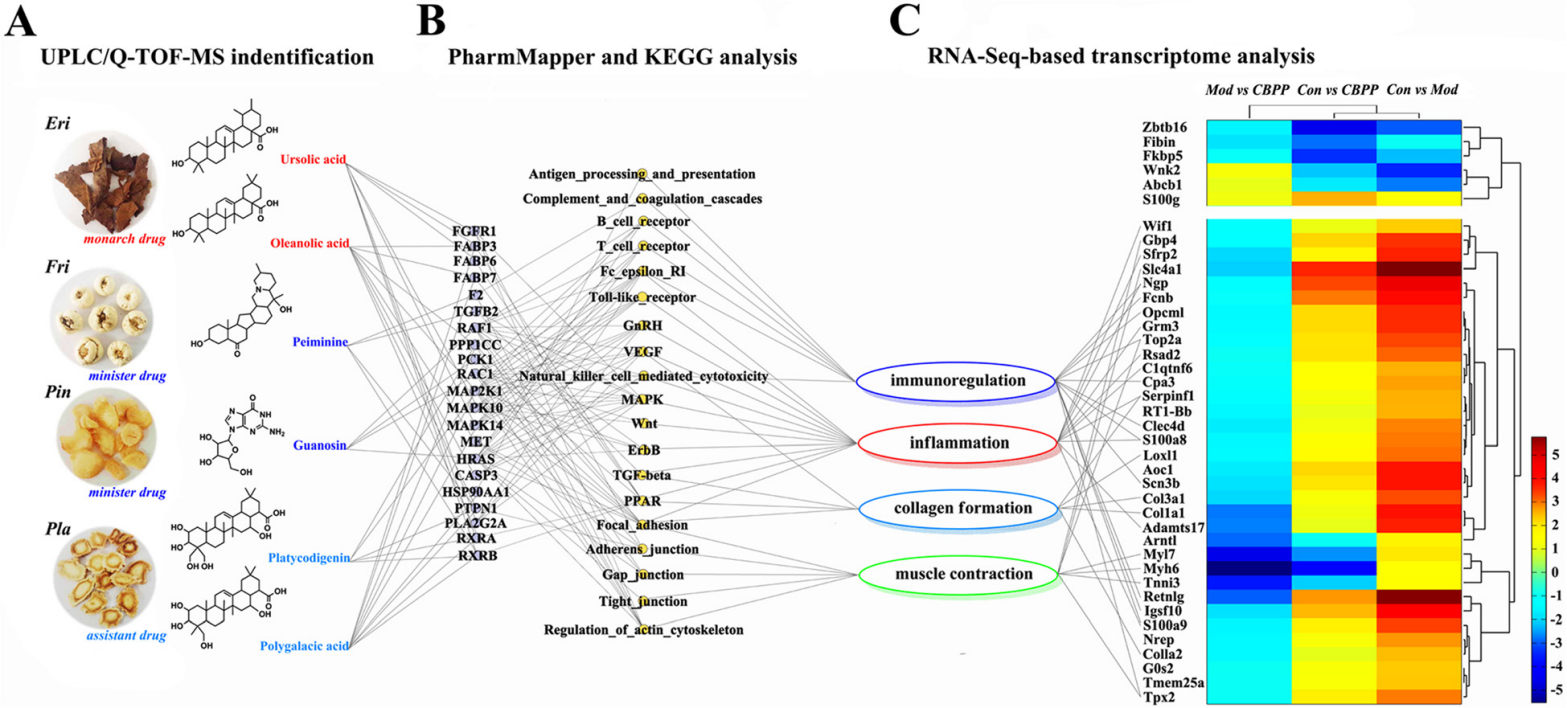
Pathway prediction and RNA-Seq analysis of CBPP. **a** Structures of five representative active compounds that were identified by UPLC/Q-TOF-MS; **b** Main targets and pathways as analyzed by PharmMapper and KEGG, respectively (network analysis); **c** RNA-Seq-based transcriptome analysis by clustering and the functional classification of 34 up-regulated genes

To further predict the targets and pathways, six representative ingredients were selected from the four herbs (Fig. 4a). These ingredients were ursolic acid and oleanolic acid from ***Eri***, peiminine from ***Fri***, platycodigenin and polygalacic acid from ***Pla***, and guanosine from ***Pin***. All of the predicted targets and pathways are shown in Additional file 1: Figure S7, and those screened from bioinformatics analysis are shown in Fig. 4b. This resulted in the identification of 15 pathways that were related to immunity or inflammation, including focal adhesion, mitogen activated protein kinase (MAPK), peroxisome proliferators-activated receptors (PPAR), toll-like receptors, transforming growth factor (TGF)-beta, ErbB, GnRH, Wnt, natural killer (NK) cell-mediated cytotoxicity, vascular endothelial growth factor (VEGF), Fc epsilon RI, antigen processing and presentation, B cell receptors, T cell receptors and complement and coagulation cascades. Among these pathways, four were related to collagen, including TGF-beta, focal adhesion, VEGF and PPAR signaling pathways; five were related to smooth muscle contraction, including adherens junction, gap junction, tight junction, actin cytoskeleton and focal adhesion.

### RNA-Seq analysis of lung tissues

The effects of CBPP-M on LPS-induced chronic bronchitis were further analyzed by RNA-Seq at the genetic level. The standards were set with a false discovery rate (FDR) ≤ 0.001 and |log2Ratio| ≥ 1. Differentially expressed genes were screened under these standards in Con *vs* Mod, Con *vs* CBPP-M and Mod *vs* CBPP-M. A total of 40 genes were screened out and then clustered according to the log2Ratio. The detailed data of the log2Ratio in the cluster are shown in Additional file 3: Table S2. As shown in Fig. 4c, 34 genes were up-regulated and six genes were down-regulated in the Con *vs* Mod. Most of differentially regulated genes were up-regulated in the Mod. However, in the CBPP-M, these were obviously down-regulated. Of the 34 up-regulated genes, 28 were associated with collagen formation, muscle contraction, inflammation or immunoregulation. Col1a1, Col1a2, Col3a1, Loxl1 and Serpinf1 are associated with collagen synthesis. Myh6, Myl7, Tnni, Scl4a1, Gbp4, Top2a and Tpx2 are associated with muscle contraction. Ten differentially expressed genes are associated with inflammation: S100a8, S100a9, Ngp, Rsad2, Clqtnf6, Wif1, Sfrp2, Grm3, Adamts17 and Serpinf1. Additionally, 12 genes are associated with immunity: Fcnb, Clec4d, Cpa3, Rectnlg, Igsf10, Scn3b, S100a8, S100a9, Ngp, Top2a, Tpx2 and Opcml.

Proline-glycine-proline (PGP) are extracellular peptides fragments found in collagen. Matrix metalloproteinase (MMP)-9 is the main enzyme involved in this procedure. The protein degradation products and enzymes were elevated in the sputum. Aerosolized LPS administered to mice has been shown to increase neutrophils and N-α-PGP levels in the airway [18]. PGP are chemoattractants in chronic neutrophilic inflammation associated with IL-8 and with recruiting inflammatory cells to the lung. In addition, collagen could trigger the VEGF and TGF-beta pathways. Gene expression profiling of the lung from chronic obstructive pulmonary disease patients revealed that Col6a3 and Serpinf1 were associated with emphysema severity [19]. Col1a1, Col1a2 and Col3a1 are involved in collagen I and III formation, and Loxl1 is usually active in collagen substrates. Our data show that these gene expression levels were noticeably decreased after CBPP-M treatment. Therefore, intervention with CBPP not only remitted airway inflammation but also reduced sputum.

Myh6 and Myl7 are components of myosin chains. Tnni confers calcium sensitivity to striated muscle actinomyosin. With increasing Ca^2+^ concentration, the interaction of actin and myosin increases and results in a cough. Chronic airway bacterial infections with symptoms of cough and wheezing have been shown to be associated with neutrophilic inflammation and high levels of IL-8 [20]. IL-8 could increase the phosphorylation of the myosin light chain and induce constriction in cystic fibrosis cells. In this paper, leukocyte numbers and IL-8 levels in BALF were increased in the Mod group. This result indicated that myosin-associated gene expression would be up-regulated. In muscle cells, the cytoskeleton and its binding proteins provide the power system for muscle contraction. Thus, the expression of the aforementioned five genes decreased in CBPP-M treatment indicates that CBPP could remit muscle contraction, arrest cough and relieve asthma.

In addition, S100a8 and S100a9 are calcium-binding proteins that have anti-microbial activity. NF-kappa-B could up-regulate the transcription of these genes. These genes promote tubulin polymerization, phagocyte migration and the infiltration of granulocytes at sites of wounding. Microtubule dynamics change with macrophage migration [21]. Ngp is a neutrophilic granule protein that increases expression and is indicative of inflammation. Wif1 and Sfrp2 are related to the Wnt signaling pathway in the regulation of inflammation. Treatment with CBPP could down-regulate the expression of these genes, demonstrating anti-inflammatory and immune-regulatory function.

### Network pharmacological action for CBPP

**Fig. 5:**
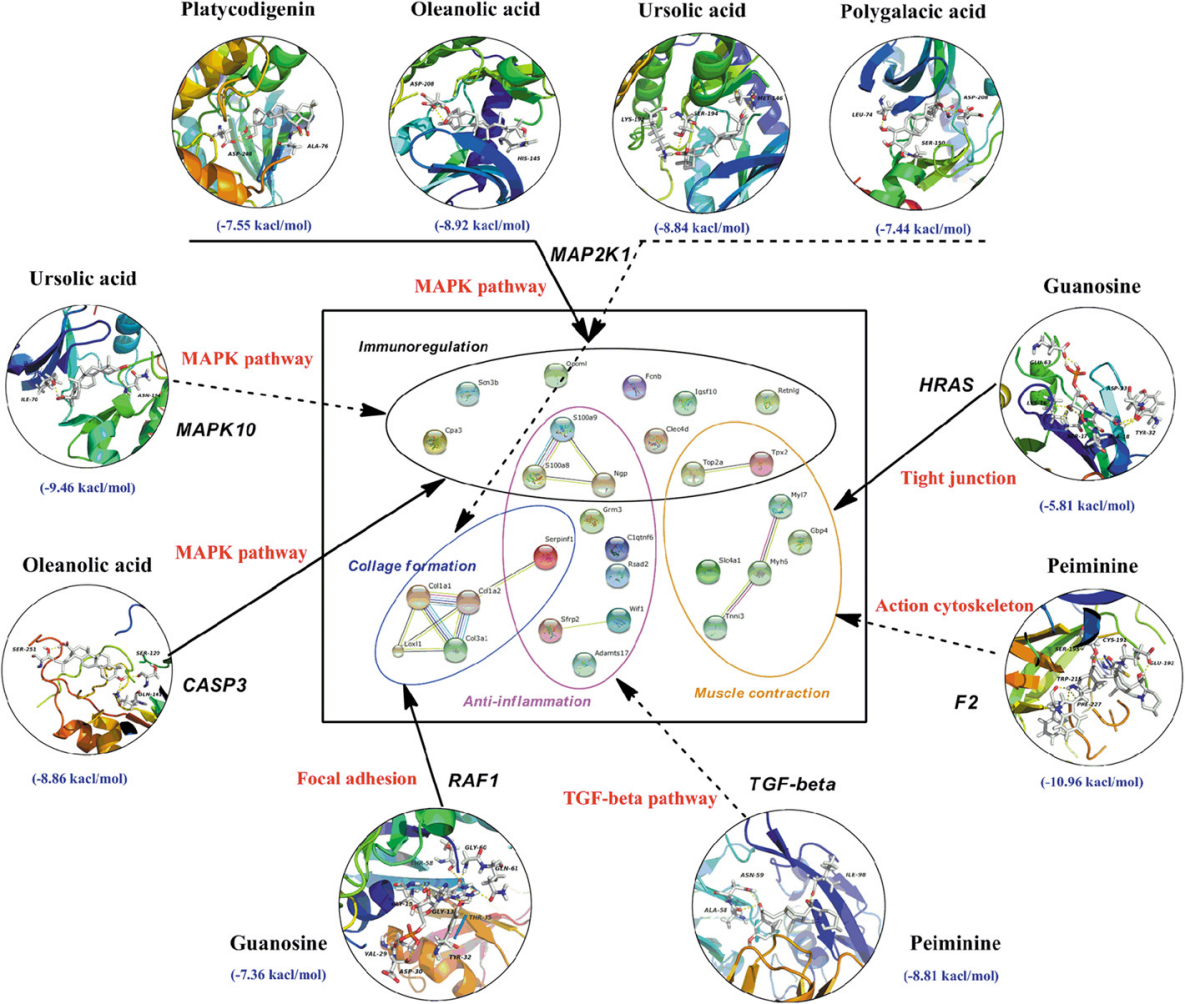
Network pharmacology analysis through the protein interaction of differentially expressed genes and protein targets and signaling pathway of representative ingredients of CBPP. The solid line represents an interaction directly confirmed through experiments or the literature, and the dotted line represents interactions indirectly speculated through docking or the literature

Airway inflammation is a complex network with many signaling pathways. Modern research has demonstrated that toll-like receptors, VEGF, MAPK, PPAR and the TGF-beta signal pathway are activated in the development of airway inflammation. In the central part of Fig. 5, the protein interaction of the above-mentioned 28 down-regulated genes was further analyzed using String 9.1. Remarkably, five protein interaction subgroups, including 15 genes, were described with ligatures. Functional proteins were assigned to four different functional categories according to collagen formation, muscle contraction, inflammation and immunoregulation. Virtual docking validation of predicted targets, which was performed using AutoDock 4.0, further showed the binding energy and detailed interaction information for the active ingredients and their related protein targets.

Ursolic acid, oleanolic acid, platycodigenin and polygalacic acid are typical pentacyclic triterpenoid compounds from ***Eri*** and ***Pla***. In this paper, these compounds were found to reduce inflammation and bind to the MAP2K1 target (PDB: 3EQF) [22]. The binding energy was -8.84, -8.92, -7.55 and -7.44 kcal/mol for the four compounds, respectively. In addition, ursolic acid also binds to the MAPK10 target (PDB: 3V6R; -9.46 kcal/mol), and oleanolic acid could bind to the CASP3 target (PDB: 3KJF; -8.86 kcal/mol). According to the KEGG analysis, all of the targets participate in the MAPK pathway. Surprisingly, ursolic acid and oleanolic acid have been found to down-regulate the phosphorylation of extracellular signal-regulated kinase (ERK)1/2 and p38 and suppress the MAPK signaling pathway [23]. In addition, the expression of MMP-9, a target gene of NF-kappa B, could be eliminated by ursolic acid in TNF-alpha-induced rat C6 glioma cell invasion [24]. Platycodin D inhibits cell invasion by reducing MMP-9 enzyme activity and potently suppressing the phosphorylation of ERK, p38, and JNK [25]. Therefore, ***Eri*** and ***Pla*** could resolve phlegm associated with inflammation by affecting the MAPK pathway.

Peiminine from ***Fri*** has been generally thought to be a cough suppressant in traditional Chinese herbal medicine. As shown in Fig. 4, F2 (PDB: 3LDX) and TGF-beta2 (PDB: 4KXZ) are its potential targets, with a binding energy of -10.96 and -8.81 kcal/mol, respectively. These interactions affect the regulation of the actin cytoskeleton and the TGF-beta pathway. In COPD, inflammation was largely driven by IL-8, TNF-alpha and TGF-beta, then cytokine and chemokine properties increased cough and airway remodeling [26]. Our results were consistent with the finding that peiminine can decrease the levels of TGF-beta, ERK1/2, and NF-kappa B in lung tissues [27].

HRAS (PDB: 4EFL; -5.81 kcal/mol) and RAF1 (PDB: 3CU8; -7.36 kcal/mol) are regarded as latent targets for guanosine. The two targets participate in focal adhesion and the MAPK pathway. These two pathways are role in reducing sputum and relieving inflammation (Fig. 4). HRAS also affect tight junctions, which are related to muscle contraction [28]. As a result, guanosine had anti-inflammatory effects and reduced sputum levels through MAPK and the focal adhesion-signaling pathway and might relieve cough through HRAS targeting.

## Conclusions

In this paper, a novel approach of global chemomics integrated systems biology was established to define the multi-component and multi-target mechanism of action of the CBPP dropping pill. Based on global chemomics identified by UPLC/Q-TOF-MS, 6 typical components, including ursolic acid, oleanolic acid, peiminine, platycodigenin, polygalacic acid, and guanosine from four herbal medicines, were predicted to affect various targets and pathways. To further validate these results, RNA-Seq-based transcriptomics was applied to a rat model of chronic bronchitis. The relevance between the therapeutic effect and effective compounds focused on immunoregulation, anti-inflammation, the inhibition of collagen formation and the regulation of muscle contraction. As stated by the TCM theory, ***Pla*** (containing platycodigenin and polygalacic acid) assists the monarch drug ***Eri*** (containing ursolic acid and oleanolic acid) to reduce inflammation and resolve phlegm by affecting the MAPK pathway; ***Fri*** (containing peiminine) as minister drug relieved cough symptoms by actin cytoskeleton and TGF-beta pathway; ***Pin*** (containing guanosine) another minister drug reduced sputum and implemented immunoregulatory actions through the MAPK and focal adhesion pathways. A holistic regulatory network of CBPP was generated for pulmonary disease treatment.

## Additional files

Additional file 1: **The quality of each herb and CBPP extract, and the targets and pathway predicted by PharmMapper and KEGG.** (DOC 4286 kb)

Additional file 2: Table S1. MS data in (±) ESI modes and the identification results for the CB extraction. (DOC 106 kb)

Additional file 3: Table S2. Differentially expressed genes screened in Con *vs* Mod, Con *vs* CB-M and Mod *vs* CB-M, and the detail number of log2Ratio in each group. (DOC 57 kb)

## Abbreviations

*Eri*: *Eriobotrya japonica* (Thunb.) Lindl.
*Fri*: *Fritillaria usuriensis* Maxim.
*Pla*: *Platycodon grandiflorum* (Jacq.) A. DC.
*Pin*: *Pinellia ternata* (Thunb.) Makino.
*Men*: *Mentha haplocalyx* Briq.
CBPP: *Eriobotrya japonica – Fritillaria usuriensis* dropping pills (ChuanbeiPipa dropping pills)
TCM: Traditional Chinese medicine
UPLC: High-quality ultra-performance liquid chromatography
Q-TOF-MS: Quadrupole time-of-flight mass spectrometry
RNA-Seq: RNA-Sequence
GO: Gene ontology
GA: Genetic algorithm
LPS: Lipopolysaccharides
BCG: Bacillus calmette-guerin
Dex: Dexamethasone
KEGG: Kyoto encyclopedia of genes and genomes
Con: Control group
Mod: Model group
PBS: Phosphate-buffered saline
BALF: Bronchoalveolar lavage fluid
H&E: Hematoxylin and eosin
TNF: Tumor necrosis factor
IL: Interleukin
MAPK: Mitogen activated protein kinase
TGF: Transforming growth factor
PPAR: Peroxisome proliferators-activated receptors
VEGF: Vascular endothelial growth factor
NK: Natural killer
FDR: False discovery rate
PGP: Proline-glycine-proline
MMP: Matrix metallo proteinase
ERK: Extracellular signal-regulated kinase
