# Supplementary material for: An integrated global chemomics and system biology approach to analyze the mechanisms of the traditional Chinese medicinal preparation Eriobotrya japonica – Fritillaria usuriensis dropping pills for pulmonary diseases
Source: BMC Complement Altern Med. 2016 Jan 8;16:4. doi: 10.1186/s12906-015-0983-y (PMC4705596; doi:10.1186/s12906-015-0983-y)
Supplement: Supplementary file 1 — The quality of each herb and CBPP extract, and the targets and pathway predicted by PharmMapper and KEGG. (DOC 4286 kb) [file 12906_2015_983_MOESM1_ESM.doc]

**Additional file 1**

**Plant Materials**

Eriobotrya  japonica – Fritillaria usuriensis dropping pills (ChuanbeiPipa dropping pills, CBPP) consists of four main medicinal components: Eriobotrya japonica (Thunb.) Lindl (***Eri***), [Fritillaria usuriensis Maxim.](http://www.theplantlist.org/tpl1.1/record/kew-306940) (***Fri***), Pinellia ternata (Thunb.) Makino (***Pin***) and Platycodon grandiflorum (Jacq.) A.DC. (***Pla***). The choice of marker compounds for each herb was guided by the Chinese Pharmacopoeia (2010 version) and the main active components described in the literature. Ursolic acid, peiminine, guanosine and platycodin D were chosen as marker compounds for ***Eri***, ***Fri***, ***Pin*** and ***Pla***, respectively. Herbal medicinal products and herbal preparation were declared as follows.

***Eri*** (Lot No. 1304763191) was provided by No.6 TCM Factory of Zhongxin Pharmaceuticals (Tianjin, China). The part of ***Eri*** used was the leaf. ***Eri***, 1 g, was dissolved in 75% aqueous ethanol solution, 10 mL, and sonicated for 30 min. After centrifugation at 6000 rpm for 10 min, the supernatant was filtered through a membrane filter (0.45 µm) and subjected to HPLC analysis. The extraction ratio of ***Eri*** was 22%. Ursolic acid, chosen as a marker compound, was purchased from Yifang Technology Co. (Tianjin, China) and was dissolved in methanol. An Agilent 1260 HPLC instrument equipped with an Agilent 1290 DAD detector (Agilent, USA) and Luna-C18 (250 mm × 4.6 mm, 5 μm) column (Phemomenex, USA) was used for this analysis. The injection volume was 20 μL at a concentration of 1 mg/mL ursolic acid and 20 μL of ***Eri*** extract. The mobile phase consisted of 0.1% (v/v) formic acid solution (I) and acetonitrile (II) at a flow rate of 1.0 mL/min. A gradient program was used as follows: 0 min, 5% B; 10 min, 15% B; 15 min, 25% B; 24 min, 45% B; 30 min, 80% B; 42 min, 100% B; 42–48 min, 100% B. The DAD detector scanned from 200 nm to 400 nm, and the monitor wavelength was set to 210 nm. The analytical methods have been validated. The calibration curves exhibited good linearity with wide linearity ranges. The limit of detection (LOD) was tested under a signal-to-noise ratio (S/N) of 3/1. The RSD values of repeatability were below 5%. The recovery test was conducted by adding accurate amounts of standard compounds. The retention time of ursolic acid was 38.27 min. The amount of ursolic acid in ***Eri*** (crude drug) was 9.24 mg/g. The 210 nm and 3D UV chromatograms of ursolic acid and the ***Eri*** extract are provided in Addition figure 1.


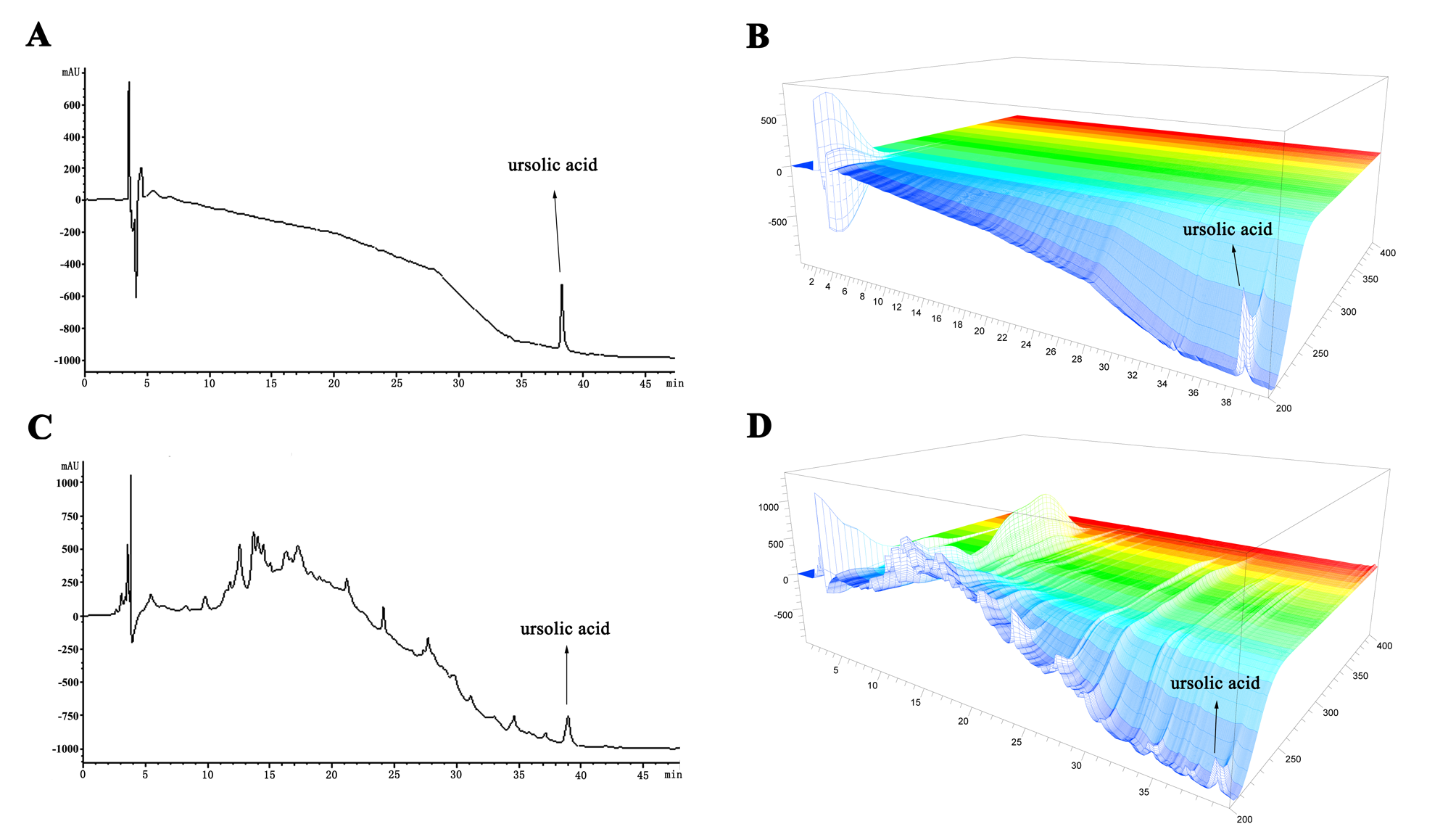


**Figure S1 Quality control of *Eri*.** (A) HPLC chromatogram of ursolic acid: monitor wave length was 210 nm, retention time was 38.27 min; (B) 3D-HPLC chromatogram of ursolic acid; (C) HPLC chromatogram of the ***Eri*** extract: monitor wave length was 210 nm, retention time was 38.86 min; (D) 3D-HPLC chromatogram of the ***Eri*** extract.

***Fri*** (Lot No. 1300304271) was provided by No.6 TCM Factory of Zhongxin Pharmaceuticals (Tianjin, China). The part of ***Fri*** used was the bulbus. ***Fri***, 1 g, was dissolved in a 75% aqueous ethanol solution, 10 mL, and sonicated for 30 min. After centrifugation at 6000 rpm for 10 min, the supernatant was filtered through a membrane filter (0.45 µm) and subjected to UPLC/Q-TOF-MS analysis. The extraction ratio of ***Fri*** was 7.8%. Peiminine, chosen as a marker compound, was purchased from Yifang Technology Co. (Tianjin, China) and was dissolved in methanol. Peiminine had no remarkable ultraviolet absorption, so it was quantified using a Waters Q-TOF Premier. A UPLC System (Waters, USA) equipped with a photodiode array detector was used. An Acquity BEH C18 column (2.1 × 100 mm, 1.7 µm; Waters, USA) was used for the separation. The injection volume was 5 μL at a concentration of 0.1 mg/mL peiminine and 5 μL of ***Fri*** extract. The mobile phase consisted of 0.1% (v/v) formic acid solution (I) and acetonitrile (II) at a flow rate of 0.4 mL/min. A gradient program was used as follows: 0 min, 2% B; 13 min, 30% B; 16 min, 50% B; 25 min, 80% B; 28 min, 100% B; 28–30 min, 100% B. The column temperature was 30 °C. Accurate mass measurements were collected using a Q-TOF Premier with an electrospray ionization system (Waters, USA). The electrospray capillary voltage was 3.0 kV for the positive mode. The sample cone voltage was 30 V. The nebulization gas was 600 L/h at 350 °C. The cone gas was 50 L/h, and the source temperature was 110 °C. The Q-TOF Premier acquisition rate was 0.1 s, with a 0.02 s inter-scan delay. The instrument was operated with the first resolving quadrupole in a wide pass mode (50 - 2,500 Da). The analytical methods have been validated. The base peak of peiminine in positive ESI mode was m/z 430.3320 and confirmed to be [M+H]^+^, and the retention time was 9.88 min. The amount of Peiminine in ***Fri*** (crude drug) was 0.21 mg/g. The total ion current chromatograms in positive ESI mode of peiminine and ***Fri*** extract are provided in Addition figure 2.


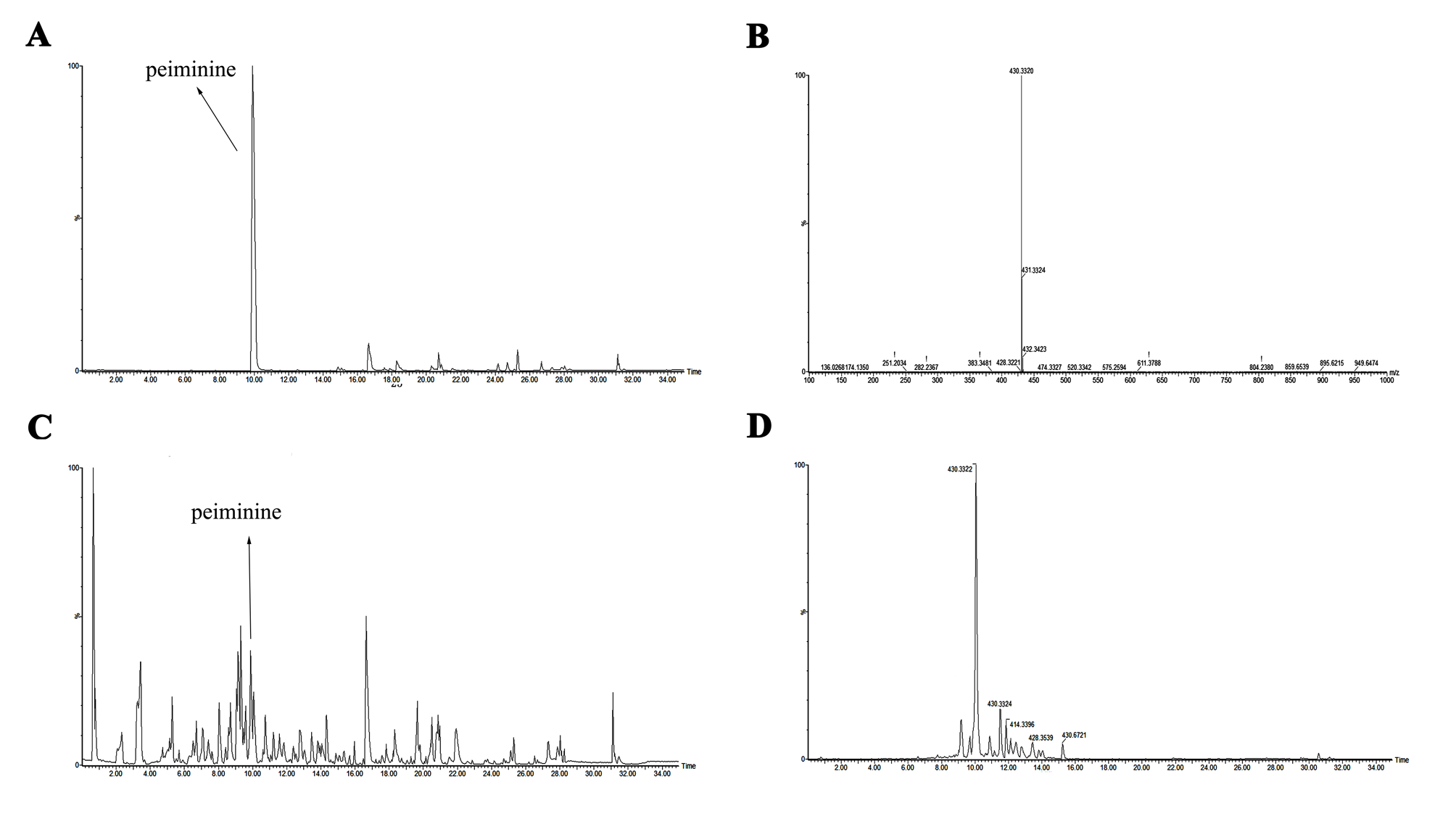


**Figure S2 Quality control of *Fri.*** (A) The total positive mode ion chromatogram of peiminine: retention time was 9.88 min,  with a measured molecular weight of 430.3320.; (B) Mass information of peiminine: The base peak in positive ESI mode was m/z 430.3320 and confirmed to be [M+H]^+^; (C) The total positive mode ion chromatogram of the ***Fri*** extract: retention time of peiminine was 9.98 min, with a measured molecular weight of 430.3322; (D) Ion chromatogram of the molecular weight 430.33 from Addition figure 2(C).

***Pin*** (Lot No. 1304227003) was provided by No.6 TCM Factory of Zhongxin Pharmaceuticals (Tianjin, China). The part of ***Pin*** used was the tuber. ***Pin***, 1 g, was dissolved in 75% aqueous ethanol solution, 10 mL, and sonicated for 30 min. After centrifugation at 6000 rpm for 10 min, the supernatant was filtered through a membrane filter (0.45 µm) and subjected to the HPLC analysis. The extraction ratio of ***Pin*** was 3.5%. Guanosine, chosen as a marker compound, was purchased from Yifang Technology Co. (Tianjin, China) and was dissolved in ultrapure water. An Agilent 1260 HPLC instrument equipped with an Agilent 1290 DAD detector (Agilent, USA) and Luna-C18 (250 mm × 4.6 mm, 5 μm) column (Phemomenex, USA) was used for this analysis. The injection volume was 20 μL at a concentration of 1 mg/mL guanosine and 20 μL of ***Pin*** extract. The mobile phase consisted of 0.1% (v/v) formic acid solution (I) and acetonitrile (II) at a flow rate of 1.0 mL/min. A gradient program was used as follows: 0 min, 5% B; 10 min, 15% B; 15 min, 25% B; 24 min, 45% B; 30 min, 80% B; 42 min, 100% B; 42–48 min, 100% B. The DAD detector scanned from 200 nm to 400 nm, and the monitor wavelength was set to 254 nm. The analytical methods have been validated. The retention time of guanosine was 5.49 min. The amount of guanosine in ***Pin*** (crude drug) was 0.06 mg/g. The 254 nm and 3D UV chromatograms of guanosine and the ***Pin*** extract are provided in Addition figure 3.


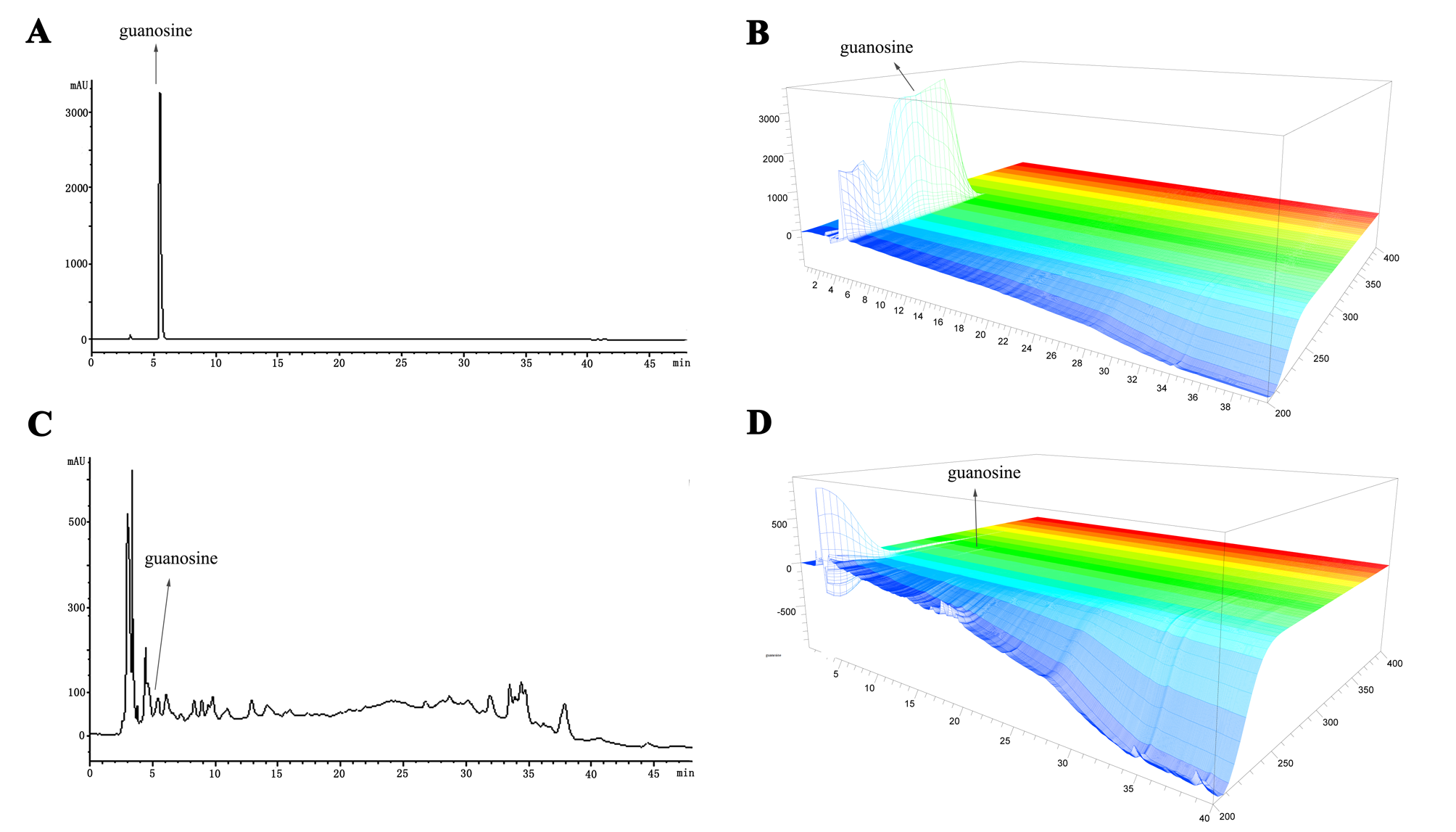


**Figure S3 Quality control of *Pin*.** (A) HPLC chromatogram of guanosine: monitor wave length was 254 nm, retention time was 5.49 min; (B) 3D-HPLC chromatogram of guanosine; (C) HPLC chromatogram of the ***Pin*** extract: monitor wave length was 254 nm, retention time was 5.78 min; (D) 3D-HPLC chromatogram of the ***Pin*** extract.

***Pla*** (Lot No. 1302083283) was provided by No.6 TCM Factory of Zhongxin Pharmaceuticals (Tianjin, China). The part of ***Pla*** used was the root. ***Pla***, 1 g, was dissolved in 75% aqueous ethanol solution, 10 mL, and sonicated for 30 min. After centrifugation at 6000 rpm for 10 min, the supernatant was filtered through a membrane filter (0.45 µm) and subjected to HPLC analysis. The extraction ratio of ***Pla*** was 17%. Platycodin D, chosen as a marker compound, was purchased from Yifang Technology Co. (Tianjin, China) and was dissolved in methanol. An Agilent 1260 HPLC instrument equipped with an Agilent 1290 DAD detector (Agilent, USA) and Luna-C18 (250 mm × 4.6 mm, 5 μm) column (Phemomenex, USA) was used for this analysis. The injection volume was 20 μL at a concentration of 1 mg/mL platycodin D and 20 μL of ***Pla*** extract. The mobile phase consisted of 0.1% (v/v) formic acid solution (I) and acetonitrile (II) at a flow rate of 1.0 mL/min. A gradient program was used as follows: 0 min, 5% B; 10 min, 15% B; 15 min, 25% B; 24 min, 45% B; 30 min, 80% B; 42 min, 100% B; 42–48 min, 100% B. The DAD detector scanned from 200 nm to 400 nm, and the monitor wave length was set to 210 nm. The analytical methods have been validated. The retention time of platycodin D was 22.05 min. The amount of platycodin D in ***Pla*** (crude drug) was 4.13 mg/g. The 210 nm and 3D UV chromatograms of platycodin D and the ***Pla*** extract are provided in Addition figure 4.


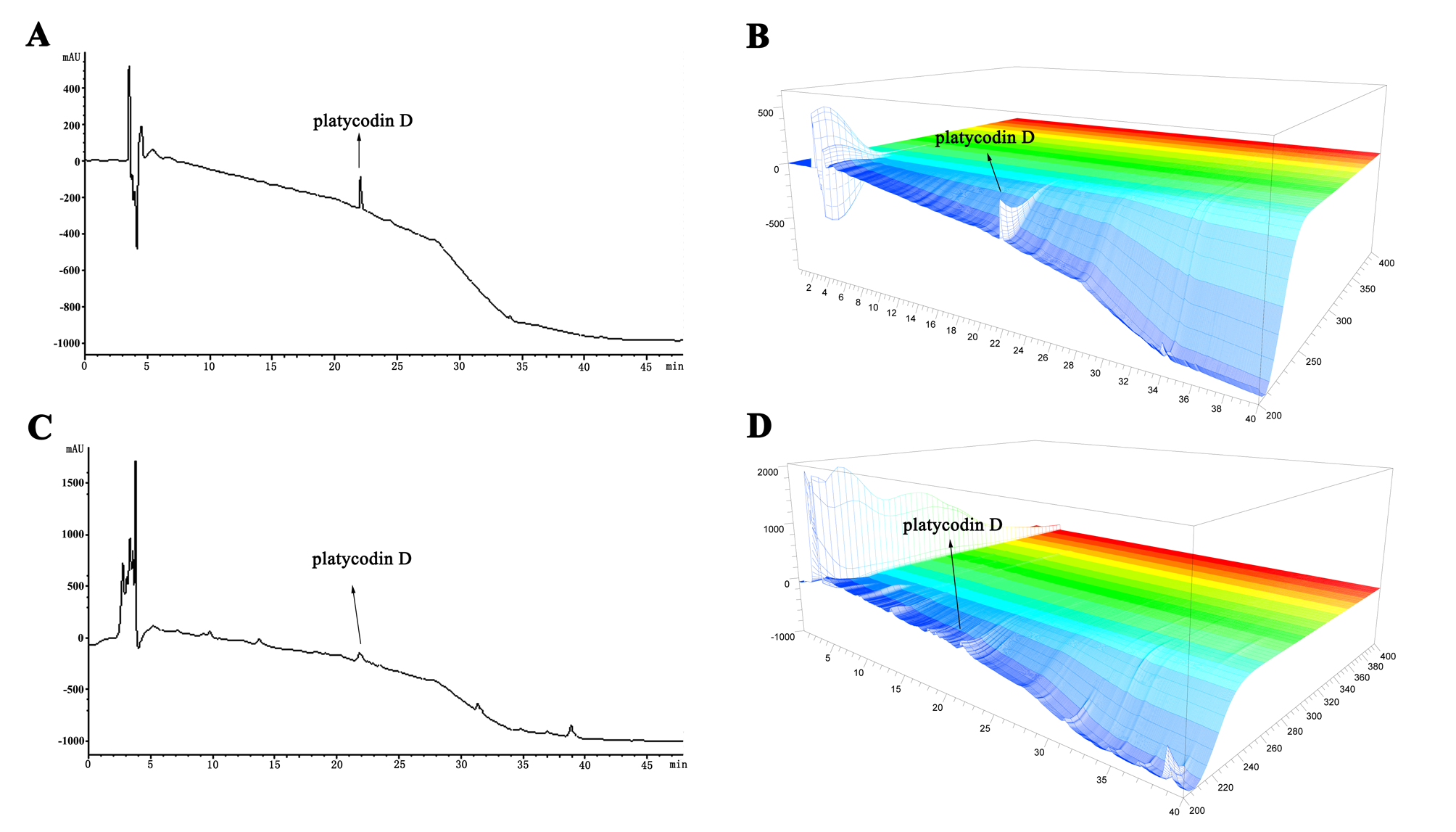


**Figure S4 Quality control of *Pla*.** (A) HPLC chromatogram of platycodin D: monitor wave length was 210 nm, retention time was 22.05 min; (B) 3D-HPLC chromatogram of platycodin D; (C) HPLC chromatogram of the ***Pla*** extract: monitor wave length was 210 nm, retention time was 21.95 min; (D) 3D-HPLC chromatogram of the ***Pla*** extract.

The CBPP extract was provided by No.6 TCM Factory of Zhongxin Pharmaceuticals (Tianjin, China). CBPP extract, 100 mg, was dissolved in a 75% aqueous ethanol solution, 10 mL, and sonicated for 30 min. After centrifugation at 6000 rpm for 10 min, the supernatant was filtered through a membrane filter (0.45 µm) and subjected to analysis. Quantitative methods of marker compounds were the same for each herbal extract. The injection volume was 20 μL in HPLC analysis and 5 μL in UPLC/Q-TOF-MS analysis. The contents of ursolic acid, peiminine, guanosine and platycodin D in the herbal preparation were 15.3 mg/g, 1.44 mg/g, 0.51 mg/g and 5.71 mg/g, respectively. The UV and ion chromatograms of the CBPP extract are provided in Addition figure 5 and Addition figure 6, respectively.


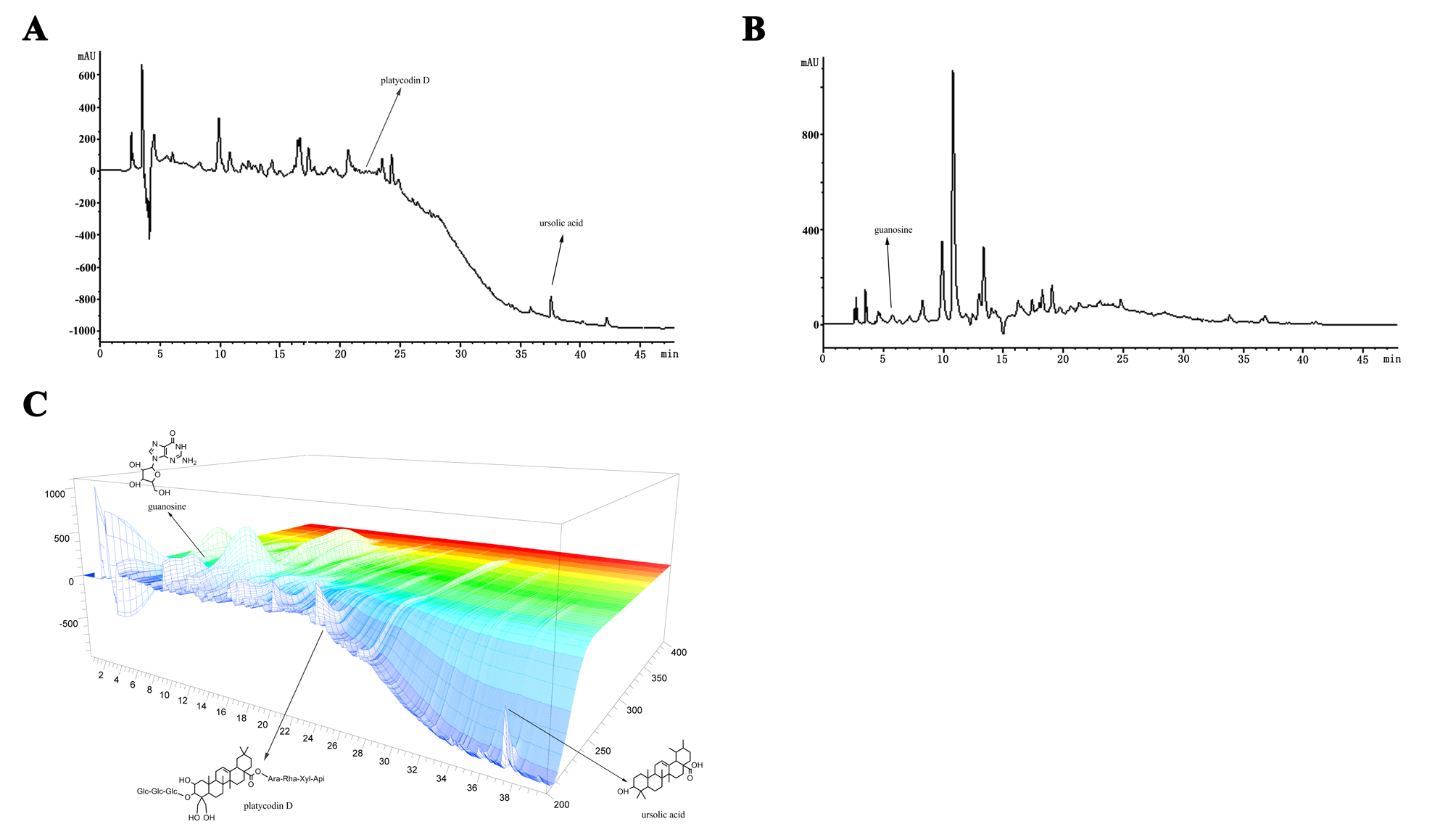


**Figure S5 Quality control of the CBPP extract by HPLC.** (A) HPLC chromatogram of the CBPP extract: monitor wave length was 210 nm, retention time of platycodin D was 22.09 min, retention time of ursolic acid was 37.83 min; (B) HPLC chromatogram of the CBPP extract: monitor wavelength was 254 nm, retention time of guanosine was 5.79 min; (C) 3D-HPLC chromatogram of the CBPP extract.


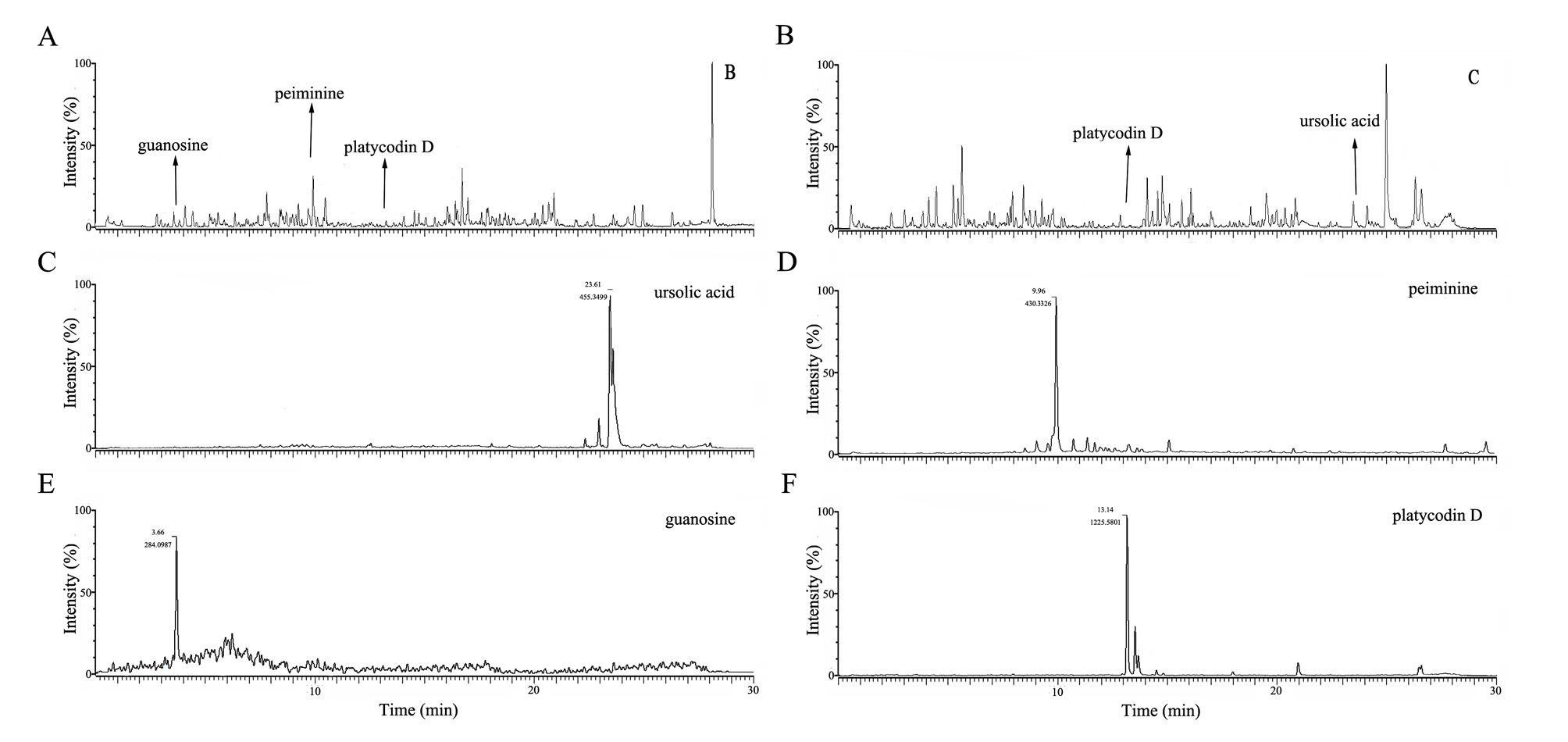


**Figure S6 Identification and quality control of the CBPP extract by UPLC/Q-TOF-MS.** (A) The total positive mode ion chromatogram of CBPP; (B) The total negative mode ion chromatogram of CBPP; (C) Ion chromatogram of the molecular weight 455.35 from Addition figure 6 (B) and the compound with a retention time 23.61 min was confirmed to be ursolic acid; (D) Ion chromatogram of the molecular weight 430.33 from Addition figure 6 (A) , and the compound with a retention time 9.96 min was confirmed to be peiminine; (E) Ion chromatogram of the molecular weight 284.09 from Addition figure 6 (A), and the compound with a retention time 3.66 min was confirmed to be guanosine; (F) Ion chromatogram of the molecular weight 1225.58 from Addition figure 6 (A), and the compound with a retention time 13.14 min was confirmed to be platycodin D.

**Target and pathway prediction of active ingredients**

In order to [further](app:ds:farther) predict the target and pathway, six representative ingredients, including ursolic acid，oleanolic acid, peiminine, platycodigenin，polygalacic acid, and guanosine were selected from the four herbs. A total of 64 targets and 94 pathway were predicted from the PharmMapper and KEGG, respectively (Addition figure 7), and screened ones from bioinformatics analysis were shown in manuscript.


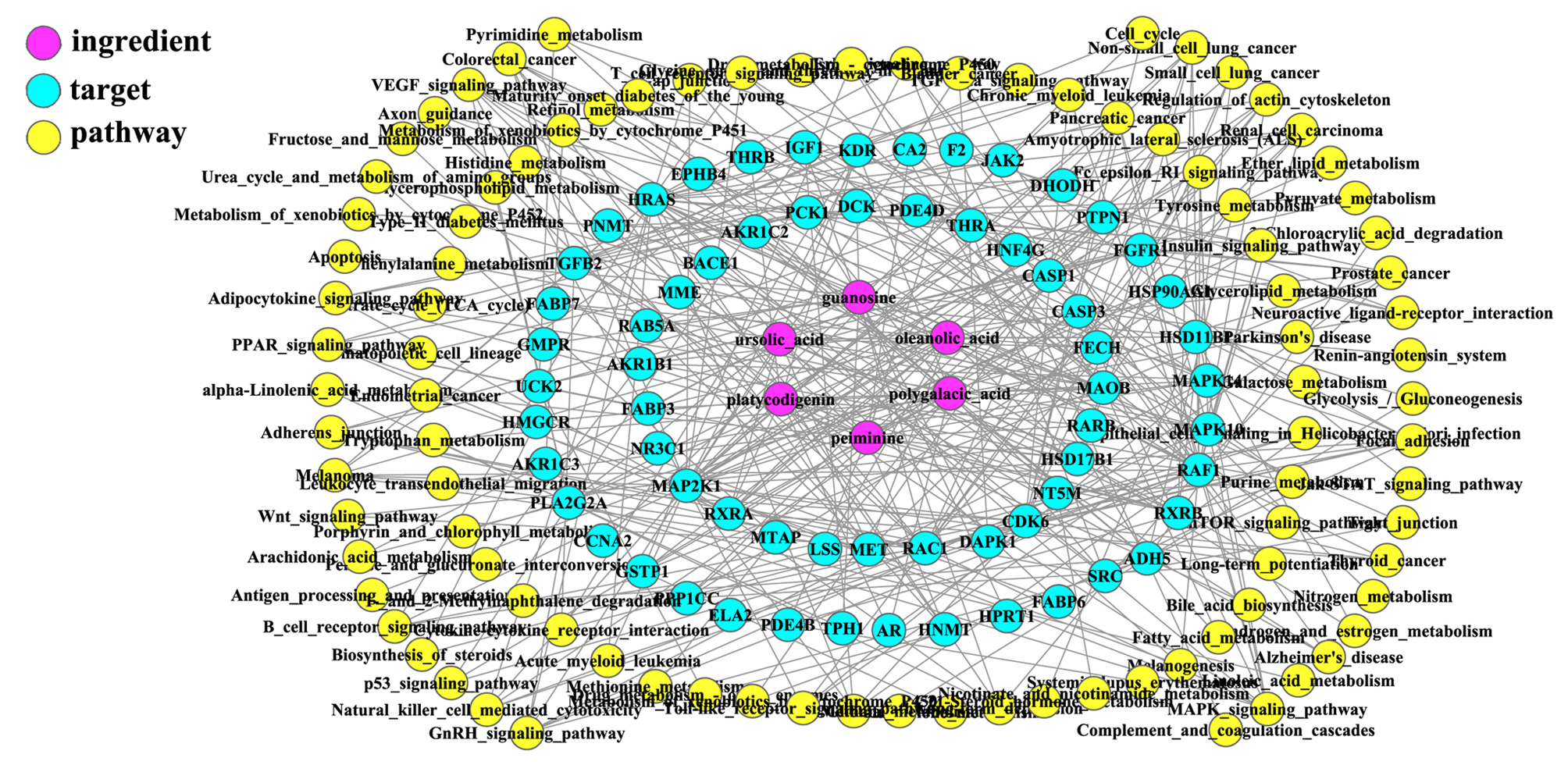


**Figure S7 Six ingredients were selected from the four different herbs in CB to** [**farther**](app:ds:farther) **predict target and pathway.** Targets and pathway were predicted by PharmMapper database and KEGG, respectively.
