# Supplementary material for: An integrated global chemomics and system biology approach to analyze the mechanisms of the traditional Chinese medicinal preparation Eriobotrya japonica – Fritillaria usuriensis dropping pills for pulmonary diseases
Source: BMC Complement Altern Med. 2016 Jan 8;16:4. doi: 10.1186/s12906-015-0983-y (PMC4705596; doi:10.1186/s12906-015-0983-y)
Supplement: Supplementary file 2 — MS data in (±) ESI modes and the identification results for the CB extraction. (DOC 106 kb) [file 12906_2015_983_MOESM2_ESM.doc]

**Additional file 2: Table S1**

MS data in (±) ESI modes and the identification results for the CB extraction.

| Peak No. | RT (min) | Mode | MS (*m*/*z*) | Composition | Error (ppm) | Identification | Herbal source |
| --- | --- | --- | --- | --- | --- | --- | --- |
| 1 | 1.17 | N | 117.0196 | C_4_H_6_O_4_ | 6.837 | succinic acid | ***Fri*** |
| 2 | 2.47 | N | 153.0179 | C_7_H_6_O_4_ | -5.882 | protocatechuic acid | ***Pin*** |
| 3 | 2.97 | P | 195.1132 | C_10_H_14_N_2_O_2_ | -1.025 | cyclo(prolylproline) | ***Pin*** |
| 4 | 3.26 | P | 245.0787 | C_9_H_12_N_2_O_6_ | 5.304 | uridine | ***Pin*** |
| 5 | 3.66 | P | 284.0987 | C_10_H_13_N_5_O_5_ | -2.816 | guanosine | ***Pin*** |
| 6 | 4.17 | P | 197.1291 | C_10_H_16_N_2_O_2_ | 0.507 | cyclo(prolylvalyl) | ***Pin*** |
| 7 | 4.56 | P | 166.1242 | C_10_H_15_NO | 6.020 | ephedrine | ***Pin*** |
| 8 | 4.62 | P | 464.3341 | C_27_H_45_NO_5_ | -7.538 | pinpeimine A | ***Fri*** |
| 9 | 5.04 | P | 478.3182 | C_27_H_43_NO_6_ | 2.718 | pinpeimine C | ***Fri*** |
| 10 | 5.69 | N | 431.0977 | C_21_H_20_O_10_ | -0.232 | apigenin-7-O-glucoside | ***Pla*** |
| 11 | 7.01 | N | 609.1418 | C_27_H_30_O_16_ | -6.238 | quercetin-7-O-rutinoside | ***Pla*** |
| 12 | 7.33 | P | 446.3669 | C_28_H_47_NO_3_ | 7.841 | pingbeinine | ***Fri*** |
| 13 | 7.42 | N | 463.087 | C_21_H_20_O_12_ | -1.512 | quercetin-7-O-glucoside | ***Pla*** |
| 14 | 8.03 | P | 446.3269 | C_27_H_43_NO_4_ | -0.224 | imperialineN-oxide | ***Fri*** |
| 15 | 8.67 | N | 447.0945 | C_21_H_20_O_11_ | 4.026 | luteolin-7-O-glucoside | ***Pla*** |
| 16 | 9.04 | N | 447.0965 | C_21_H_20_O_11_ | 8.499 | kaempferol-3-O-glucoside | ***Eri*** |
| 17 | 9.06 | P | 428.3132 | C_27_H_41_NO_3_ | -7.705 | peimisine | ***Fri*** |
| 18 | 9.46 | N | 457.0765 | C_22_H_18_O_11_ | -1.313 | epigallocatechin-3-gallate | ***Eri*** |
| 19 | 9.96 | P | 430.3326 | C_27_H_43_NO_3_ | 1.162 | peiminine | ***Fri*** |
| 20 | 10.61 | P | 416.3137 | C_26_H_41_NO_3_ | -6.726 | pingbeinone | ***Fri*** |
| 21 | 11.12 | N | 301.0318 | C_15_H_10_O_7_ | -9.966 | quercetin | ***Eri*** |
| 22 | 11.69 | P | 401.1428 | C_18_H_24_O_10_ | -4.986 | regaloside A | ***Fri*** |
| 23 | 12.37 | P | 414.3365 | C_27_H_43_NO_2_ | -1.689 | cordiline | ***Fri*** |
| 24 | 12.63 | N | 739.1679 | C_39_H_32_O_15_ | 2.165 | cinchonainIIb | ***Eri*** |
| 25 | 12.92 | P | 1093.5331 | C_52_H_84_O_24_ | -9.145 | deapioplatycodin D | ***Pla*** |
| 26 | 13.14 | P | 1225.5801 | C_57_H_92_O_28_ | -4.243 | platycodin D | ***Pla*** |
| 27 | 13.66 | P | 1135.5538 | C_54_H_86_O_25_ | 0.176 | platycoside B | ***Pla*** |
| 28 | 13.91 | P | 1267.5996 | C_59_H_94_O_29_ | 2.919 | platycodin C | ***Pla*** |
| 29 | 14.22 | N | 413.1195 | C_22_H_22_O_8_ | -9.924 | picropodophyllin | ***Fri*** |
| 30 | 14.81 | N | 867.1403 | C_43_H_32_O_20_ | -0.692 | theaflavin 3 | ***Eri*** |
| 31 | 14.81 | N | 821.4161 | C_39_H_66_O_18_ | -1.217 | loquatifolin A | ***Eri*** |
| 32 | 14.87 | N | 329.2333 | C_18_H_34_O_5_ | 1.519 | pinellic acid | ***Pin*** |
| 33 | 15.58 | N | 503.3352 | C_30_H_48_O_6_ | -4.172 | 19-hydroxyasiatic acid | ***Eri*** |
| 34 | 15.7 | P | 1371.6461 | C_63_H_102_O_32_ | 2.114 | polygalacin D2 | ***Pla*** |
| 35 | 16.44 | N | 575.4309 | C_35_H_60_O_6_ | -0.521 | daucosterol | ***Pin*** |
| 36 | 16.59 | N | 441.3709 | C_30_H_50_O_2_ | -5.438 | betulin | ***Pla*** |
| 37 | 16.68 | P | 373.1275 | C_20_H_20_O_7_ | -3.216 | tangeritin | ***Pla*** |
| 38 | 16.98 | P | 343.1379 | C_16_H_22_O_8_ | -4.080 | coniferin | ***Pin*** |
| 39 | 16.99 | P | 361.1654 | C_20_H_24_O_6_ | 0.831 | lariciresinol | ***Pin*** |
| 40 | 18.16 | P | 359.1484 | C_20_H_22_O_6_ | -3.063 | 5-O-Methylhierochin D | ***Pin*** |
| 41 | 18.55 | N | 485.3385 | C_30_H_46_O_5_ | 8.242 | 2,3,19-trihydroxy-ursa-5,12-dien-28-oic acid | ***Eri*** |
| 42 | 18.84 | N | 469.3568 | C_31_H_50_O_3_ | -2.983 | mathylursolate | ***Eri*** |
| 43 | 19.36 | N | 723.1696 | C_39_H_32_O_14_ | -2.489 | kaempferol3-O-α-L-(2',4'-di-Z-p-coumaroyl)-ramnoside | ***Eri*** |
| 44 | 19.57 | N | 633.3865 | C_39_H_54_O_7_ | -0.789 | 3-coumaroylrotundic acid | ***Eri*** |
| 45 | 19.62 | N | 385.3465 | C_27_H_46_O | -1.298 | cholesterol | ***Pin*** |
| 46 | 19.82 | N | 471.3475 | C_30_H_48_O_4_ | 0.212 | maslinic acid | ***Eri*** |
| 47 | 20.02 | N | 471.3478 | C_30_H_48_O_4_ | 0.849 | corosolic acid | ***Eri*** |
| 48 | 20.87 | N | 515.2487 | C_25_H_40_O_11_ | -0.970 | eriojaposide B | ***Eri*** |
| 49 | 22.48 | N | 563.1193 | C_29_H_24_O_12_ | 0.533 | theaflavin 1 | ***Eri*** |
| 50 | 22.96 | N | 647.3995 | C_40_H_56_O_7_ | 7.260 | 3-O-trans-feruloyleuscaphic acid | ***Eri*** |
| 51 | 23.49 | N | 455.3531 | C_30_H_48_O_3_ | 1.318 | oleanolic acid | ***Eri*** |
| 52 | 23.61 | N | 455.3499 | C_30_H_48_O_3_ | -5.710 | ursolic acid | ***Eri*** |
| 53 | 24.14 | N | 289.0709 | C_15_H_14_O_6_ | -1.038 | catechin | ***Eri*** |
| 54 | 24.62 | P | 323.2599 | C_20_H_34_O_3_ | 4.022 | 3,16,17-trihydeoxykaurane | ***Fri*** |
| 55 | 25.02 | N | 279.2329 | C_18_H_32_O_2_ | 1.791 | linoleic acid | ***Pin*** |
| 56 | 26.34 | N | 255.2313 | C_16_H_32_O_2_ | -4.310 | hexadecanoic acid | ***Pin,Eri*** |
| 57 | 26.61 | N | 281.2477 | C_18_H_34_O_2_ | -1.422 | oleic acid | ***Pin*** |
| 58 | 26.62 | P | 265.2558 | C_18_H_32_O | 10.179 | 9,12,15-octadecatrien-1-ol | ***Eri*** |
