# Supplementary material for: An integrated global chemomics and system biology approach to analyze the mechanisms of the traditional Chinese medicinal preparation Eriobotrya japonica – Fritillaria usuriensis dropping pills for pulmonary diseases
Source: BMC Complement Altern Med. 2016 Jan 8;16:4. doi: 10.1186/s12906-015-0983-y (PMC4705596; doi:10.1186/s12906-015-0983-y)
Supplement: Supplementary file 3 — Differentially expressed genes screened in Con vs Mod, Con vs CB-M and Mod vs CB-M, and the detail number of log2Ratio in each group. (DOC 57 kb) [file 12906_2015_983_MOESM3_ESM.doc]

**Additional file 3: Table S2**

**Differentially expressed genes screened in Con *vs* Mod, Con *vs* CB-M and Mod *vs* CB-M, and the detail number of log2Ratio in each group.**

| Gene Symbol | log2Ratio  (Con *vs* Mod) | log2Ratio  (Con *vs* CB-M) | log2Ratio  (Mod *vs* CB-M) |
| --- | --- | --- | --- |
| Col1a1 | 3.948419 | 1.188696 | -2.75972 |
| Adamts17 | 3.733394 | 1.025869 | -2.70753 |
| Igsf10 | 4.396743 | 2.50468 | -1.89206 |
| Sfrp2 | 3.720829 | 1.745331 | -1.9755 |
| Aoc1 | 3.9105 | 2.205446 | -1.70505 |
| Scn3b | 3.90596 | 2.036585 | -1.86938 |
| Tpx2 | 2.958329 | 1.908829 | -1.0495 |
| Rsad2 | 3.117985 | 2.102433 | -1.01555 |
| Top2a | 3.350646 | 2.062635 | -1.28801 |
| Grm3 | 3.602185 | 2.181848 | -1.42034 |
| Opcml | 3.621294 | 2.181848 | -1.43945 |
| Gbp4 | 3.543291 | 2.230758 | -1.31253 |
| S100a9 | 3.399145 | 1.866601 | -1.53254 |
| RT1-Bb | 2.558599 | 1.053311 | -1.50529 |
| Col1a2 | 2.484882 | 1.000731 | -1.48415 |
| Serpinf1 | 2.584164 | 1.260302 | -1.32386 |
| Nrep | 2.78263 | 1.315026 | -1.4676 |
| Wif1 | 2.334462 | 1.088012 | -1.24645 |
| Cpa3 | 2.699086 | 1.518883 | -1.1802 |
| C1qtnf6 | 2.580766 | 1.468257 | -1.11251 |
| Tmem255a | 2.389379 | 1.358862 | -1.03052 |
| G0s2 | 2.347896 | 1.331062 | -1.01683 |
| Clec4d | 2.902751 | 1.210485 | -1.69227 |
| S100a8 | 2.965524 | 1.454798 | -1.51073 |
| Loxl1 | 3.057666 | 1.630211 | -1.42746 |
| Col3a1 | 3.304369 | 1.331697 | -1.97267 |
| Fcnb | 4.092755 | 2.964112 | -1.12864 |
| Ngp | 4.723864 | 3.341789 | -1.38208 |
| Retnlg | 5.706522 | 2.76681 | -2.93971 |
| Slc4a1 | 5.760845 | 3.683617 | -2.07723 |
| S100g | 1.412047 | 2.565177 | 1.15313 |
| Arntl | 1.817848 | -1.04723 | -2.86508 |
| Tnni3 | 1.555264 | -2.09117 | -3.64643 |
| Myl7 | 2.000149 | -2.59367 | -4.59382 |
| Myh6 | 1.505817 | -4.03262 | -5.53844 |
| Zbtb16 | -2.95733 | -4.52248 | -1.56516 |
| Fibin | -1.04979 | -2.82852 | -1.77873 |
| Fkbp5 | -2.24877 | -3.36043 | -1.11166 |
| Abcb1b | -2.7341 | -1.70864 | 1.025463 |
| Wnk2 | -3.46991 | -2.18061 | 1.289303 |
